# A practical approach to optic nerve crush in the mouse

**Published:** 2012-07-27

**Authors:** Justin P. Templeton, Eldon E. Geisert

**Affiliations:** Department of Ophthalmology, Hamilton Eye Institute, The University of Tennessee Health Science Center, Memphis, TN

## Abstract

Our goal is to provide an instructional resource to help others wishing to use the optic nerve crush (ONC) as an experimental procedure. The process is described beginning with the anesthesia, followed by positioning of the mouse, the surgery itself, and post-surgical care. We analyzed the effect of ONC on retinal blood flow, using optical coherence tomography doppler. This procedure produces a consistent loss of cells in the ganglion cell layer, using whole mounts of retina stained with TO-PRO-3. An instructional video is presented that demonstrates a simple surgical approach to effectively crush the optic nerve of the mouse.

## Introduction

The retina is a central nervous system (CNS) structure with one white matter tract, the optic nerve, connecting the retinal ganglion cells to their targets within the brain. The isolation of these axons from any surrounding gray matter provides a unique opportunity to create a pure axonal injury by crushing or cutting the nerve. Some people have used this system as a model for glaucomatous damage, a disease that many believe is triggered by axonal damage at the optic nerve head [1]. Optic nerve crush (ONC) has advantages over other methods, such as optic nerve transections, for it is relatively mild and does not interrupt ocular blood flow. The ONC is particularly useful as a simple synchronous approach for examining ganglion cell injury in a large number of mouse strains [2]. This experimental model produces an insult with many of the same molecular changes that occur in murine models of glaucoma where there is both an induced and/or intrinsic elevation of intraocular pressure [3-7]. The present paper describes a simple and effective method of crushing the optic nerve of the mouse and also offers a step-by-step instructional video.

## Methods

### Mice used

The procedure described can be used with any common strain of mouse. For the instructional video demonstration, we used a DBA/2J mouse because the anatomic structures are more visible on a pigmented mouse than the albino BALB/cByJ mouse used for RGC counting. In the Results section we describe data obtained from eight male, BALB/cByJ retinas from mice 60–90 days old. All procedures were in compliance with institutional guidelines and with the ARVO statement for the Use of Animals in Ophthalmic and Vision Research. The Institutional Animal Care and Use Committee at the University of Tennessee Health Science Center approved all protocols involving the use of mice. The mice were anesthetized with a mixture of 13 mg/kg of Rompum and 87 mg/kg of Ketalar.

### Positioning the mouse

In the beginning phases of your work, an assistant should help hold the anesthetized mouse or tape can be used to restrain the mouse. It is necessary for the eye to protrude to allow access during the procedure. As the technique improves it is possible to hold the mouse with one hand and use the free hand to operate. The mouse is held under the dissecting microscope perpendicular to the operator with its nose toward the operator’s dominant hand.

### The ONC procedure

Under the binocular operating scope, a small incision is made with spring scissors (cat. #RS-5619; Roboz, Gaithersburg, MD) in the conjunctiva beginning inferior to the globe and around the eye temporally. Use caution as making this cut too deep can result in cutting into the underlying musculature (inferior oblique, inferior rectus muscles inferiorly or the lateral rectus temporally) or the supplying vasculature. With micro-forceps (Dumont #5/45 forceps, cat. #RS-5005; Roboz), grasp the edge of the conjunctiva next to the globe and retract it, rotating the globe nasally. This exposes the posterior aspect of the globe, allowing you to visualize the optic nerve. The exposed optic nerve is grasped approximately 1–3 mm from the globe with Dumont #N7 cross-action forceps (cat. #RS-5027; Roboz) for 10 s, with the only pressure from the self-clamping action to press on the nerve. The Dumont cross-action forceps was chosen because its spring action applied a constant and consistent force to the optic nerve. During the 10 s clamping, you should be able to see mydriasis. This observed response is variable, depending on the mouse strain and the mechanism of action of the anesthetic used. After 10 s the optic nerve is released and the forceps are removed, allowing the eye to rotate back into place. In the video to illustrate the anatomic landmarks associated with the ONC, we surgically exposed the nerve to reveal the nerve and the forceps clamping the nerve.

### Post-operative procedure

At the end of the procedure, a drop of 0.5% proparacaine hydrochloride ophthalmic solution (Falcon Pharmaceuticals, Fort Worth, TX) is administered for post-operative pain control, and a small amount of surgical lubricant (KY jelly; McNeil-PPC, Skillman, NJ) is applied to the eye to protect it from drying. The mouse is placed on a warming pad and monitored until it fully recovers from anesthesia. The mouse is monitored for post-operative complications for several days. The possible complications are infection, bleeding, and loss of muscular control; these complications are rare (less than 1%) as long as an aseptic technique is used during surgery. When monitoring for infection, begin by looking for swelling of the surgical site, protruding of the eye from posterior swelling, change in eye size, lens discoloration, or purulent drainage from the wound. Any cutaneous bleeding that does not resolve by the end of the procedure needs to be addressed so it does not result in a secondary ischemic injury to the eye.

### Immunohistochemical analysis of retinal ganglion cell loss

For the analysis of ganglion cell loss, the mice were allowed to survive for 30 days, after which they were deeply anesthetized with a mixture of 13 mg/kg Rompum and 87 mg/kg Ketalar and then perfused through the heart with saline followed by 4% paraformaldehyde in 0.1 M sodium phosphate buffer (pH 7.3). We post fixed the eyes for 24 h in 4% paraformaldehyde, then rinsed them in PBS (sodium chloride – 137 mmol/l, potassium chloride – 2.7 mmol/l, disodium phosphate – 10 mmol/l, potassium phosphate monobasic – 2.0 mmol/l, and pH 7.4) before the retinas were dissected free from the globe and held in PBS. After rinsing the retinas, four small cuts were made in each one to assist in the retinal flat mounts. While on the slide the retina was covered with TO-PRO-3 iodide (T3605; Invitrogen, Eugene, OR), diluted to 1 μM/ml in PBS, for 20 min then rinsed three times with PBS. The PBS was removed and the retina was flooded with Fluoromount-G (Cat. No. 0100–01, SouthernBiotech, Birmingham AL) and covered with a coverslip.

### Imaging the whole mount retina for cell counts

Imaging was performed at low power (4×), using the Nikon Eclipse TE2000-E (Nikon Instruments Inc., Melville, NY) confocal microscope. The area of each retina was calculated using a 1-μm scale bar and NIH ImageJ software. To determine the density of cells in a given retina, we placed a sampling grid over the 4× image of the retina in an orientation that maximized fields across all regions of the retina. We then photographed the retina at each of the intersect points in the grid at 40×. This resulted in a minimum of 14 and a maximum of 18 sampling fields per retina. The images of each field were assigned a random nonidentifying identifier, and the labeled cells were counted by an investigator blind to the study. We used the mean number of cells per field in the control and ONC retinas to determine the change in cell number within the retinal ganglion layer following ONC.

### Ultra-high resolution optical coherence tomography

To measure blood flow in the retina immediately before and after ONC, we used Doppler optical coherence tomography (OCT) on six different mice. The anesthetized mouse eyes were dilated with 1% tropicamide. The mouse was then wrapped in gauze during imaging to maintain body heat and placed in a holder with the head positioned for imaging using a bite bar. The imaging was performed using the Bioptigen ultra-high resolution OCT system and a mouse bore. The system detects light reflected from the retina to generate a cross-sectional image of the retinal layers. With the Doppler option selected while scanning, we were able to image directional blood flow.

## Results

**Figure 1:**
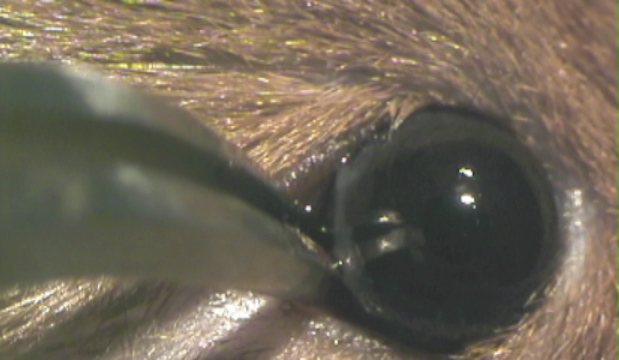
Tutorial video of the optic nerve crush procedure. This video is a tutorial of the ONC procedure beginning with a description of the instruments, then positioning the mouse, and finally the procedure itself.

The basic procedure is presented in a tutorial video (Figure 1). The video begins with a statement concerning the use of animals and compliance with regulations set forth by our university and ARVO. There is a brief summary of the instruments used in the video along with the company and catalog numbers of each. This is followed by the orientation and positioning of the mouse for the procedure and then the procedure itself. Step A: the initial incision is described and then shown; Step B: the blunt dissection and exposure of the optic nerve; Step C: the actual “crush” of the optic nerve with the curved cross-action forceps for 10 s and the exposure of the nerve to illustrate the anatomic landmarks; Step D: release of the optic nerve. The post-procedure application of topical anesthetics and surgical lubricant is shown. Finally, there is an Acknowledgment section listing funding sources.

### Blood flow in the retina

**Figure 2:**
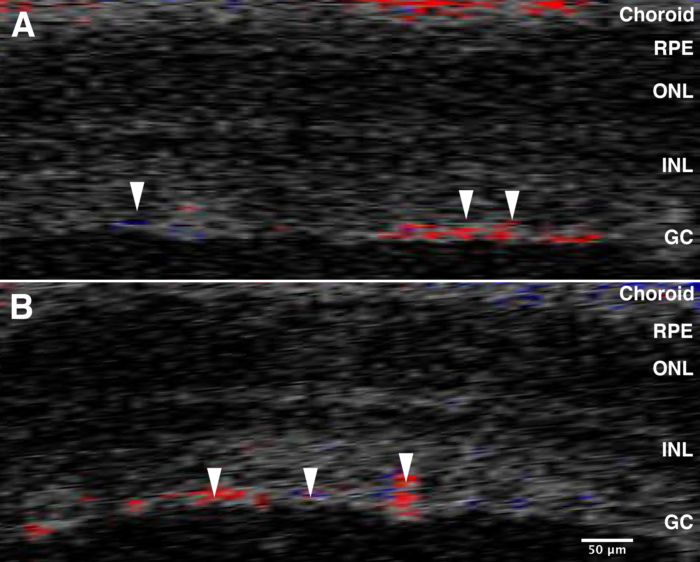
Optical coherence tomography Doppler imaging before and after optic nerve crush (ONC). OCT was used to measure blood flow using Doppler imaging. We were able to confirm that there is continued perfusion of the retina before and after ONC. Panel **A** shows the OCT Doppler image of the retina immediately before ONC, and panel **B** illustrates the blood flow immediately after ONC. The arrows point to some of the vessels present on the surface of the retina. The colors reflect the direction of blood flow; red is flow toward the transducer and blue is flow away from the transducer. These data demonstrate that in the mouse this procedure does not interrupt blood flow to the retina. The abbreviations in the figures are defined as follows: retinal pigment epithelium (RPE), outer nuclear layer (ONL), inner nuclear layer (INL), and ganglion cells (GC).

One possible result of this procedure is the interruption of the blood flow to the retina. In the mouse, unlike the human, the ophthalmic artery enters the optic nerve immediately before the lamina. It enters the retina as the central retinal artery and branches over the surface of the retina. Using the Doppler blood flow capabilities of our OCT, we were able to monitor blood flow in the retina immediately before and after ONC (Figure 2) [8]. There was no change. Thus, any changes observed in the retina due to the ONC procedure were due to damaging the axons within the optic nerve and not due to alterations in blood flow to the retina.

### Analyzing the change in retinal ganglion cell loss

**Figure 3:**
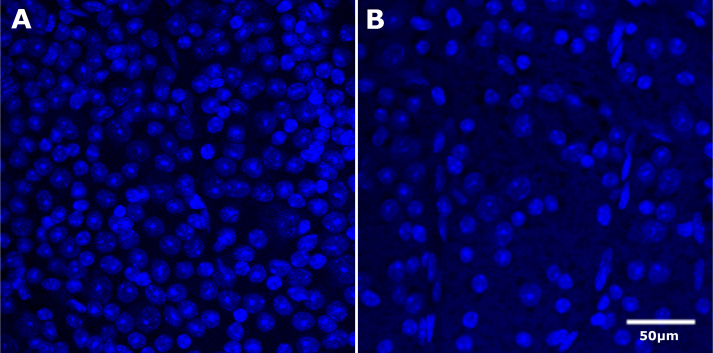
Example of TO-PRO-3 images in BALB/cByJ control and optic nerve crush (ONC) retinas. Panel **A** and panel **B** demonstrate the TO-PRO-3 staining in the BALB/cByJ control and ONC retina, respectively. Only the RGC layer cells that were not obviously endothelial cells were counted. There were four individual retinas with approximately 12 fields each, totaling 48 counted fields per control and ONC group. The scale bar is 50 µm.

**Figure 4:**
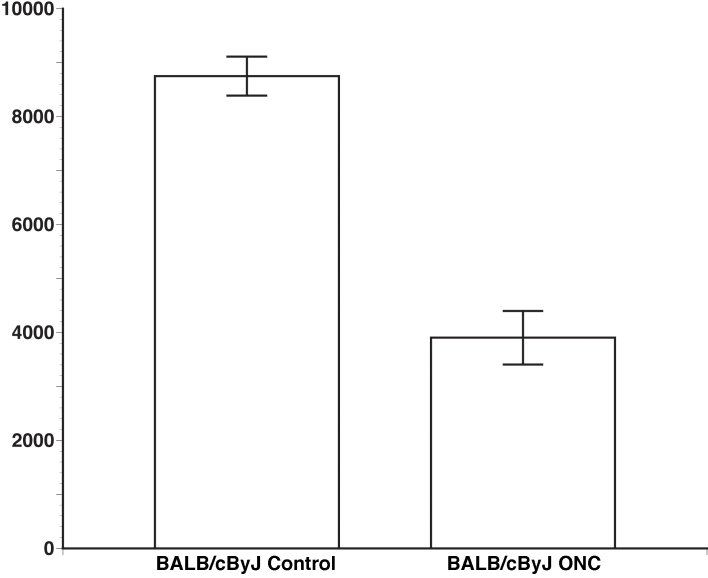
Comparison of cell number in control and optic nerve crush (ONC) retinas. The graph shows the average cell count of BALB/cByJ using TO-PRO-3 labeling. The control group has an average of 8,750 cells/mm^2^ with a standard error of 359; the ONC group has an average of 3,901 cells/mm^2^ with a standard error of 496. This illustrates a significant loss of retinal ganglion cells (p<0.01).

To define the efficacy of the ONC, cells in the ganglion cell layer of BALB/cByJ retinas were stained with TO-PRO-3 and counted in a blinded manner (Figure 3). The BALB/cByJ mouse that was used for counting has been shown to have approximately 55,000 ganglion cells within each retina [9]. TO-PRO-3 labels the total number of nucleated cells in the ganglion cell layer. Within the retinal ganglion cell layer there are ganglion cells and a considerable number of displaced amacrine cells [10]. The control retinas had an average of 8,750 cells/mm^2^ with a standard error of 359 cells/mm^2^. After ONC the number of cells decreased to 3,901 cells/mm^2^ with a standard error of 496 cells/mm^2^. These data are presented in Figure 4. There was a relatively small standard error indicating a consistent loss of retinal ganglion cells. These data demonstrate the ability of this method to produce a significant and consistent loss of retinal ganglion cells (Student *t* test, p<0.01). A loss of axons can also be observed as demonstrated by a recent publication from our research group [11].

## Discussion

ONC is a common method used to study the effect of axon injury. It can be used as a model of glaucoma or as an approach to CNS injury in general [11-14]. In the rodent, ONC is a synchronous acute approach to study the changes occurring in the cell body in an attempt to understand potential changes occurring in glaucoma, a disease that causes a slow, chronic, asynchronous death of retinal ganglion cells [2,6,15-17]. Although there are many different methods to crush the nerve, we have developed a procedure that is rapid and relatively easy. The modest improvements we have made are presented in our video (Figure 1). This instructional resource may help others wishing to use ONC and may provide a degree of uniformity to the procedure. We feel that the described procedure is simple and easy to learn.

A variety of techniques can be used to injure the optic nerve in rodents: physically transecting the nerve [18-20], using clips to crush the nerve [12,21], or using forceps to crush the nerve [13,22-24]. In addition to the studies crushing the nerve, the duration of the crush varies considerably, ranging from 3 to 5 s [13,22], 10 s [23,24], 15 s [25], to 60 s in rats [26]. The different methods and timing variations of the procedure are determined based on the animal, the injury being simulated, as well as the method of analysis post crush. Another significant difference that varies from laboratory to laboratory is the control eye, whether it is the contralateral eye or an eye from a separate control animal. We use the cross-action forceps for 10 s to maximize the effect on the retina [27,28]. Clearly the duration of the crush can be varied depending upon the specific experimental design.
